# Circulating nano-particulate TLR9 agonist scouts out tumor microenvironment to release immunogenic dead tumor cells

**DOI:** 10.18632/oncotarget.10379

**Published:** 2016-07-01

**Authors:** Yuji Kitahata, Tomohiro Kanuma, Masayuki Hayashi, Nobuyoshi Kobayashi, Koji Ozasa, Takato Kusakabe, Burcu Temizoz, Etsushi Kuroda, Hiroki Yamaue, Cevayir Coban, Takuya Yamamoto, Kouji Kobiyama, Taiki Aoshi, Ken J. Ishii

**Affiliations:** ^1^ Labotatory of Adjuvant Innovation, National Institutes of Biomedical Innovation, Health and Nutrition (NIBIOHN), Osaka, Japan; ^2^ 2nd Department of Surgery, Wakayama Medical University, Wakayama, Japan; ^3^ Laboratory of Vaccine Science, WPI Immunology Frontier Research Center (IFReC), Osaka University, Osaka, Japan; ^4^ Laboratory of Malaria Immunology, WPI Immunology Frontier Research Center, Osaka University, Osaka, Japan; ^5^ Department of Pediatrics, Yokohama City University, Yokohama, Japan

**Keywords:** CpG-ODN, β-glucan, innate immunity, immunogenic cell death, phagocytes

## Abstract

Recent evidence suggest that a β-glucan derived from mushroom Schizophyllan(SPG) complexed with a humanized TLR9 agonistic CpG DNA, K3 (K3-SPG) is a promising vaccine adjuvant that induces robust CD8 T cell responses to co-administered antigen. However, it has not been investigated whether K3-SPG alone can act as an anti-cancer immunotherapeutic agent or not. Here, we demonstrate that intravenous injection of K3-SPG, but not CpG alone, is accumulated in the tumor microenvironment and triggered immunogenic cell death (ICD) of tumor cells by local induction of type-I interferon (IFN) as well as IL-12. Resultant innate immune activation as well as subsequent tumor-specific CD8 T cell responses were contributed the tumor growth suppression. This anti-tumor effect of K3-SPG monotherapy was also confirmed by using various tumor models including pancreatic cancer peritoneal dissemination model. Taken together, nano-particulate TLR9 agonist injected intravenously can scout out tumor microenvironment to provoke local innate immune activation and release dead tumor cells into circulation that may induce broader and protective tumor antigen-specific CD8 T cells.

## INTRODUCTION

Various strategies for fighting cancer have been proposed [1, 2], some of which target the tumor microenvironment. To overcome the heterogeneity of tumor cells as well as immunosuppressive status in the microenvironment, an alternative strategy such as tumor-infiltrating macrophages and/or dendritic cells (DCs) targeting may be required for switching them from ignorant or suppressive immune milieu towards protective anti-tumor innate and adaptive immunity [3, 4]. It also has been reported that under certain condition, tumor cells undergoing programmed cell death exhibit immunogenic cell death (ICD) [5, 6], that can elicit the phagocytosis of dead cell-derived tumor antigens by DCs, resulting in the expansion of tumor antigen-specific cytotoxic T lymphocytes [7, 8].

In this study, we examined previously reported vaccine adjuvant K3-SPG [10], a nano-particulate TLR9 ligand complexed with SPG, for their anti-cancer activities because K3-SPG was composed of two known anti-cancer agents, Schizophyllan (SPG) and K3-CpG-DNA. SPG, a water soluble β-glucan derived from mushrooms, Schizophyllum commune [11], has been used clinically to treat cervical cancer in combination with irradiation in Japan [12] and known to be phagocytosed by macrophages [11]. Given that SPG has been an approved anti-cancer agent for decades, we examine whether nano-particulate TLR9 ligand complexed with SPG induce any synergistic anti-tumor effects between CpG and SPG, or whether SPG carries CpG into tumor microenvironment to induce anti-tumor immune responses without the addition of exogenous tumor antigen.

## RESULTS

### Intravenous K3-SPG injection suppress tumor growth

**Figure 1:**
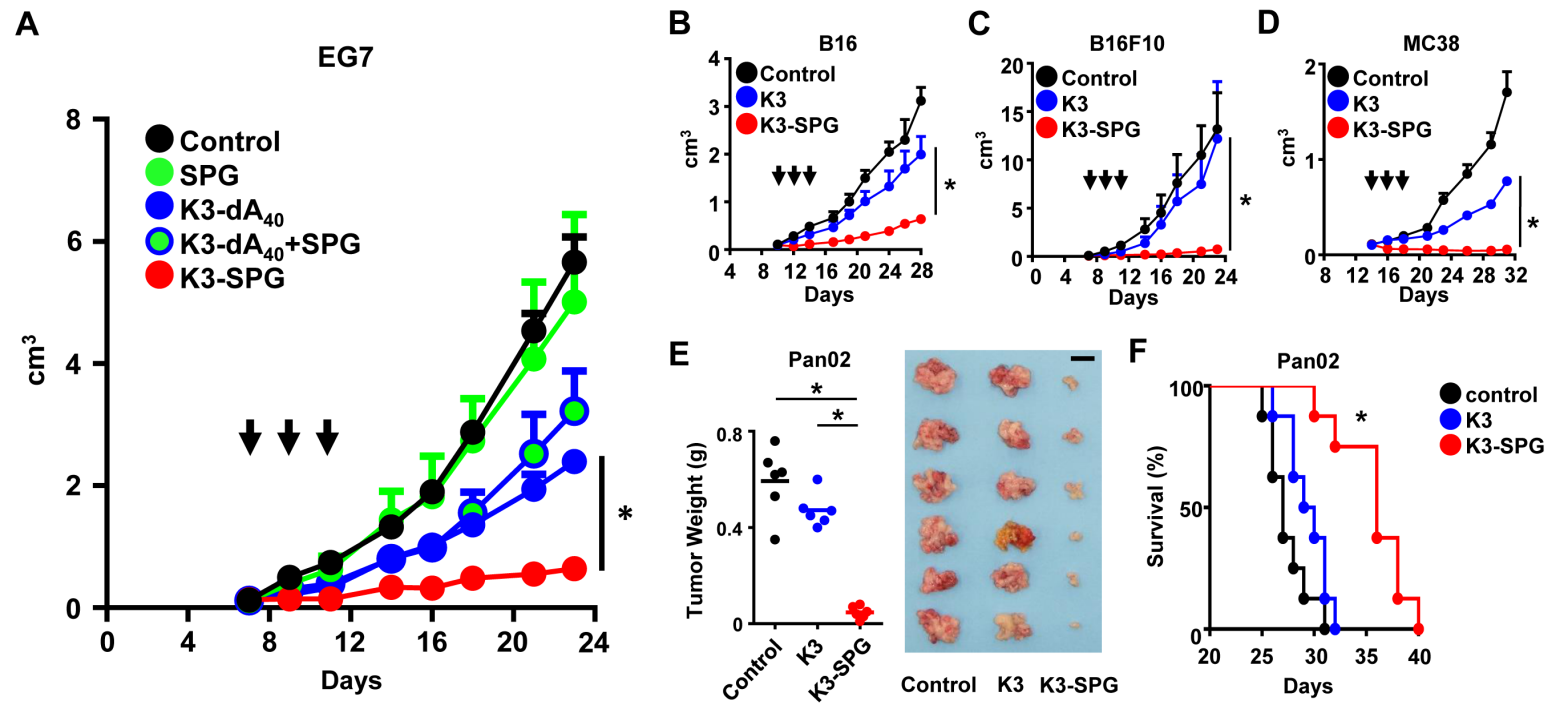
Systemic administration of K3-SPG without tumor antigen shrinks established tumor **A.** C57BL/6 mice were inoculated with EG7 subcutaneously into the right flank at day 0 and were treated with intravenous (i.v.) SPG, K3-dA_40_, K3-dA_40_+SPG, or K3-SPG complex at days 7, 9, and 11 (*n* = 4). Arrows indicate timing of therapy. (b-d) C57BL/6 mice were inoculated with B16 **B.**, B16F10 **C.** or MC38 **D.** at day 0. Tumor bearing mice were treated i.v. with PBS, K3 or K3-SPG at days 10, 12, and 14 (B16), or days 7, 9, and 11 (B16F10), or days 14, 16, and 18 (MC38). Error bars represent mean + SEM (*n* = 4). *p < 0.05 (*t*-test). **E.** C57BL/6 mice were intraperitoneally (i.p.) injected with Pan02 at day 0 and were treated i.v. with PBS, K3 or K3-SPG at days 11, 13, or 15. Tumor weight is representative at day 21. *p < 0.05 (*t*-test) (scale bars, 1 cm). **F.** C57BL/6 mice were i.p. injected with Pan02 at day 0 and were treated i.v. with PBS, K3 or K3-SPG three times. Survival rate (%) was representative (*n* = 8). *p < 0.05 (Logrank test).

The TLR9 agonist CpG DNA has been widely used as a cancer vaccine adjuvant [13-16] or an intra-tumoral mono-therapeutic agent [17]. Recently, we have developed a nanoparticulate TLR9 agonistic CpG DNA K3-(CpG hereafter) complexed with SPG, namely K3-SPG as a potent vaccine adjuvant to induce Th1 and CD8 T cell responses [10]. K3-SPG have a rod-like structure with an average diameter of 30 nm as a soluble monomeric nanoparticle, and the size is comparable to SPG itself [10]. In the current study, we investigated the effect of K3-SPG as a mono-therapeutic anti-tumor agent on an explant tumor mouse models. First, EG7 thymoma-bearing mice were established and treated 3 times (days 7, 9, and 11) with SPG, CpG (K3-dA_40_), SPG mixed with K3-dA_40_, or K3-SPG (complex), or phosphate buffered saline (PBS) as a control by intravenous (i.v.) administration. Intratumoral injection of CpG alone shrank the tumor (Suppl Figure 1) consistent with previous reports [18-21]; however, i.v. injection of CpG alone and SPG mixed with CpG had no effect on tumor size (Figure 1A). In contrast to previous reports [9], the i.v. administration of SPG alone had no effect on tumor regression (Figure 1A). By sharp contrast, K3-SPG complex reduced tumor size significantly not only when administered intra-tumorally, but also when injected intravenously (Figure 1A, Suppl Figure 1).

### K3-SPG is effective irrespectively to the origin, aggressiveness or growing environment of tumor cells

We asked whether the effect of K3-SPG is limited to the origin, tumor antigen or aggressiveness of tumor. When i.v. administration of K3-SPG after tumor transplantation, K3-SPG was also effective in suppressing the growth of other types of tumors such as melanoma (B16, B16F10), colon cancer (MC38) when compared to K3 i.v. administration (Figure 1B, 1C, 1D). We then tested the use of K3-SPG further using a more clinically relevant pancreatic cancer peritoneum dissemination model, into which intratumoral administration is technically impossible. Pancreatic cancer (Pan02) cells disseminated in the peritoneum and killed all hosts within 35 days with or without K3 treatment alone (Figure 1E, 1F). By sharp contrast, i.v. administration of K3-SPG significantly reduced the disseminated tumor mass and weight (Figure 1E), resulting in a prolonged survival rate (Figure 1F). These results suggested that i.v. administration, an uncommon route for immunostimulants, potentiated K3-SPG to be an effective mono-immunotherapeutic agent for multiple types of cancer. But, the dose escalation test of systemic administration of K3 and K3-SPG without tumor antigen in pancreatic cancer peritoneum dissemination model revealed that there is no difference of the effect of K3-SPG compared with K3, suggesting that K3-SPG has dose sparing effect (Suppl Figure 2).

### K3-SPG targets phagocytic cells in the tumor microenvironment

**Figure 2:**
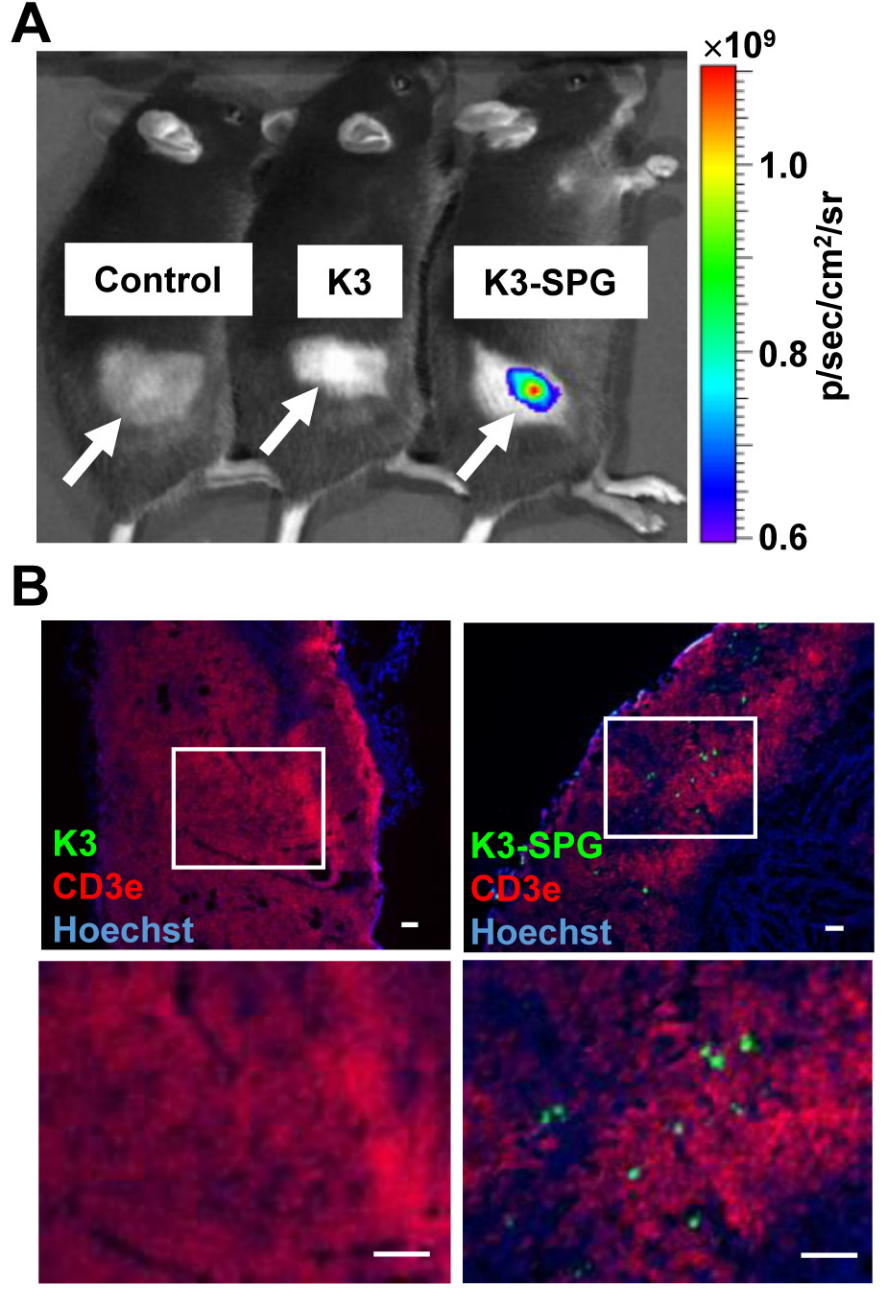
K3-SPG the tumor microenvironment **A.** Mice were inoculated s.c. with EG7 and were administrated with PBS, Alexa647-K3 i.v. or Alexa647-K3-SPG, at day 12. One hour after administration, mice were analyzed by IVIS, in which images measured in relative fluorescence were converted into physical units of surface radiance (photons/sec/cm^2^/sr). White arrows indicate tumor-inoculated areas. **B.** Frozen sections of tumor were stained by anti-CD3e antibody (red, EG7 staining) and Hoechst 33258 (blue, nucleus staining) and analyzed by fluorescent microscopy (scale bars, 100 μm).

To understand the mechanism of action by which i.v. injection of K3-SPG was so effective, we hypothesized that the nanoparticle formation of K3-SPG [10] either enhanced systemic innate immune activation and/or modified its targeting to specific tissues or cells. Indeed, SPG accumulates in tumors [9], and is a non-agonistic ligand for Dectin-1, a C-type lectin receptor expressed on phagocytes [22]. We therefore examined the *in vivo* distribution of K3-SPG. EG7 tumor-bearing mice were injected intravenously with PBS, Alexa647-K3, or Alexa647-K3-SPG. Macroscopic analysis by an *in vivo* fluorescence imaging system (IVIS) revealed K3-SPG accumulated in the tumor site in 1 hour after administration, whereas the Alexa647-K3 signal was not detected (Figure 2A). We also observed that K3-SPG was taken up by reticuloendothelial system of the liver and spleen after 1 hour of administration. At 24 hour after administration, we could not detect any signals from Alexa647-K3-SPG neither in tumor or reticuloendothelial system of the liver and spleen. This result suggested rapid clearance or degradation of K3-SPG *in vivo*. We also tested liver toxicity after K3-SPG administrations, and found no detectable liver dysfunction in terms of serum ALT and AST levels (data not shown).

**Figure 3:**
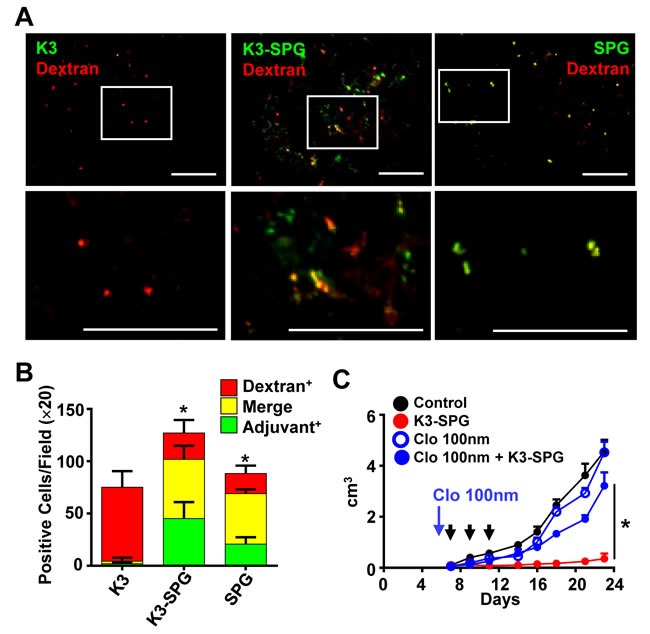
K3-SPG scouts out phagocytes in the tumor microenvironment **A.** Mice were inoculated s.c. with EG7 and were administrated with Alexa647-K3, Alexa647-K3-SPG, or FITC-SPG i.v. together with dextran-PE at day 12. One hour after injection, frozen sections of tumors were analyzed by fluorescent microscopy (scale bars, 100 μm). **B.** Green, red or merged cells were counted (10 fields from each of 3 tumors). Error bars represent mean + SD. Asterisks indicate significant differences from K3 injected merged cell numbers. **C.** Mice (*n* = 3 or 4) were inoculated s.c. with EG7 and were administrated with clodronate liposome or control liposome i.v. at day 5. Mice were injected with PBS or K3-SPG 3 times. Error bars represent mean + SEM. Arrows indicate timing of therapy. *p < 0.05 (*t*-test).

We further examined K3-SPG distribution in the tumor with immunohistochemical and microscopic analysis. The results showed that K3-SPG signal positive cells accumulated in the tumor microenvironment, but no such positive cells were observed in the K3 injected group (Figure 2B). Importantly, K3-SPG was not associated with CD3e expressing EG7 cell population, indicating that K3-SPG was taken up by non-tumor cells (Figure 2B). Instead, K3-SPG positive cells were those that engulfed TRITC-dextran (Figure 3A), while FITC-labeled SPG alone but not K3 alone was co-localized with TRITC-dextran positive cells (Figure 3A), at a comparable level to that of K3-SPG in a quantitative manner (Figure 3B). This indicated that the distribution of K3-SPG in the tumor microenvironment was controlled by SPG preferentially taken up by phagocytes. Of note, K3-SPG containing phagocytes were stained with a variety of macrophages and dendritic cell markers including CD11b, CD11c, CD169, F4/80, and MARCO, and none of these markers were dominantly co-stained with the K3-SPG containing phagocytes, suggesting that K3-SPG were taken up by a mixture of different types of tumor resident phagocytes.

### Phagocytes is required for K3-SPG-mediated anti-tumor effect

To examine the importance of phagocytes for anti-tumor effect of K3-SPG, we injected clodronate liposomes (CL) i.v. [23] to deplete phagocytic cells. After CL injection, F4/80 positive cells were depleted at least in the tumor (Suppl Figure 3). When tumor-bearing mice were injected with CL before mice were treated with K3-SPG, the K3-SPG-mediated tumor regression was completely lost (Figure 3C). These results suggested that the phagocytes and engulfment of K3-SPG by phagocytes might be essential for anti-tumor effect of K3-SPG. We performed further studies using *Dectin-1*-deficient mice to examine the contribution of receptor of SPG [24].However, Dectin-1 was not required for anti-tumor effects of K3-SPG (Suppl Figure 4). We therefore speculated that K3-SPG might passively be accumulated in the interstitial space within the tumor *via* an enhanced permeability and retention (EPR) effect [25-27], because K3-SPG forms approximately 30 nm of nano-particle.

### Intravenous K3-SPG administration released immunogenic dead tumor cells

**Figure 4:**
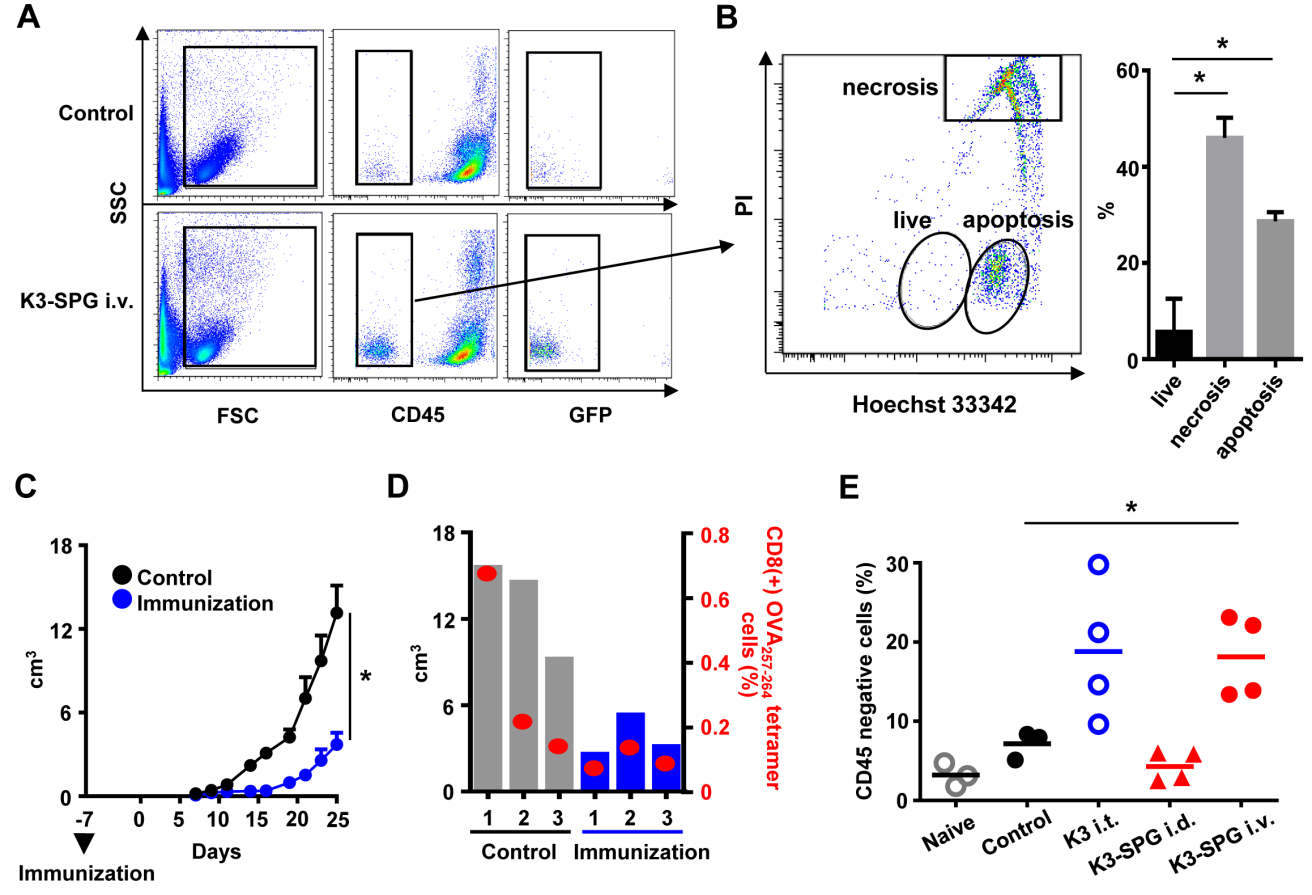
K3-SPG induces immunogenic cell death, which is essential for tumor regression **A.** GFP mice were inoculated s.c. with EG7, treated i.v. with PBS or K3-SPG at days 7, 9, and 11, and were sacrificed at day 12. Splenocytes were collected and stained by anti-CD45 antibody and cells were analyzed by flow cytometry. **B.** CD45- populations were stained by Hoechst 33342 and propidium iodide (PI) for dead cell staining and analyzed by flow cytometry. Bar graphs indicate the population of apoptotic, necrotic, and live CD45- cells. Error bars represent mean + SEM (*n* = 3). *p < 0.05 (*t*-test). **C.** Mice were immunized with or without CD45- cells. Seven days after immunization, mice were inoculated with EG7 s.c., and the size of tumors were measured (*n* = 3). Error bars represent mean + SEM. *p < 0.05 (*t*-test). **D.** Tumor volume and OVA_257-264_ specific tetramer positive CD8 T-cell numbers are represented by bar and scatter graphs, respectively, at day 25. *p < 0.05 (*t*-test). **E.** Mice were inoculated with EG7 s.c. and were treated with PBS, or K3-SPG i.v. or K3 i.t., or K3-SPG intradermally on same schedule as (A Splenocytes were collected and stained by anti-CD45 antibody and analyzed by flow cytometry. Scatter plots indicate CD45- cell populations. *p < 0.05 (*t*-test).

During the immunological analysis of tumor bearing mice after intravenous K3-SPG administration, we observed increased number of CD45 negative ‘non-immune cells’ in the spleen (data not shown). To confirm whether these cells were really tumor cells, we explanted EG7 cells into green fluorescent protein (GFP) transgenic mice, and treated them with K3-SPG at days 7, 9, and 11 after tumor cell explantation. At day 12, the spleen of GFP-mice bearing EG7 contained a large amount of both GFP and CD45 negative cells compared with the PBS-treated group, indicating these cells were derived from tumor cells (Figure 4A). Hoechst and propidium iodide staining revealed that almost all of the CD45 negative cells were dead cells undergoing either apoptosis or necrosis (Figure 4B). To determine whether the dead tumor cells were increasingly trapped in the spleen and could prime anti-tumor immune responses equivalent to ICD, we sorted and i.v. injected these CD45 negative cells into naïve mice as a tumor vaccine immunization. Then immunized and naïve mice were challenged with live EG7 cells after 7 days. Dead tumor cell-immunized mice were significantly protected against EG7 tumor challenge compared with naïve mice (Figure 4C). Of interest, the number of OVA_257-264_ specific CD8 T-cells in the spleen was inversely correlated with tumor size, suggesting that OVA_257-264_ specific CD8 T cells were not protective and instead the dead tumor cells became more immunogenic and potentially expanded protective antigens or neo-antigens (Figure 4D). Indeed, at least in our hands, effective anti-cancer therapies, such as intratumoral K3 treatment and intravenous K3-SPG treatment, but not ineffective K3-SPG treatment, are associated with higher numbers of CD45 negative tumor cells in the spleen (Figure 4E). Thus, intravenous K3-SPG administration can induce highly effective immunogenic tumor cell death, potentially broadening the tumor antigenicity.

### Innate immune activation is required for immunogenic tumor cell death

**Figure 5:**
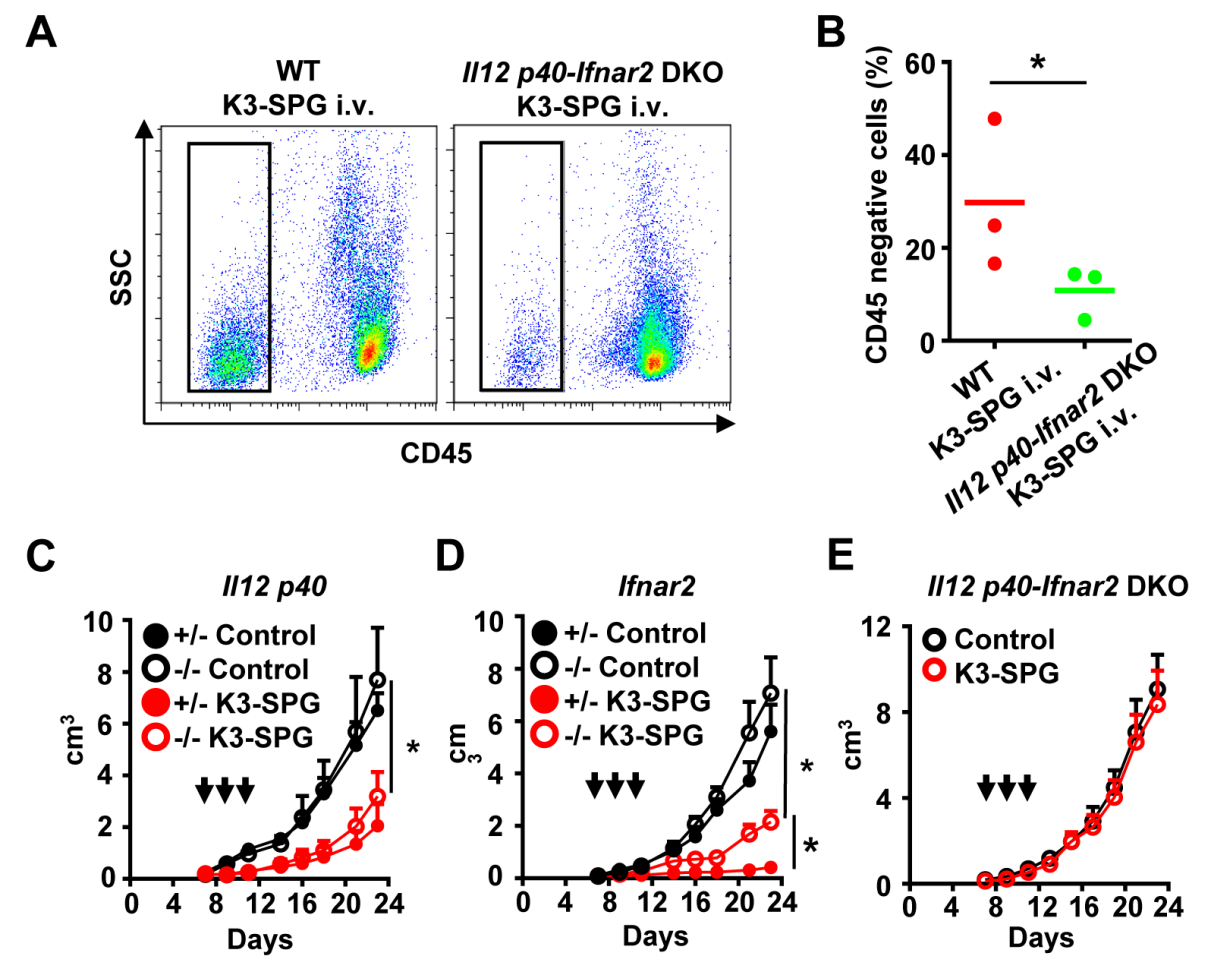
Both *IL-12* and *IFN* contributes to immunogenic cell death following intravenous K3-SPG treatment **A.** C57BL/6 mice and *Il12p40-Ifnar2* double deficient mice were inoculated s.c. with EG7 cells at day 0, treated intravenously (i.v.) with K3-SPG at days 7, 9, and 11, and then were sacrificed at day 12. Splenocytes were collected and stained by anti-CD45 antibody and then analyzed by flow cytometry. **B.** Scatter plots indicate CD45 negative cell populations. *p < 0.05 (*t*-test). (**C.**-**E.**) *Il12 p40-*, *Ifnar2-*hetero and deficient mice, and *Il12p40-Ifnar2* double deficient mice were inoculated with EG7 s.c. into the right flank at day 0 and then were treated with PBS, or K3-SPG i.v. at days 7, 9, and 11. Error bars represent mean + SEM (*n* = 4). Arrows indicate timing of therapy. *p < 0.05 (*t*-test).

We then determined whether immunogenic tumor cell death caused by intravenous K3-SPG administration was induced by innate immune activation. We focused on interleukin (IL)-12 and type-I IFN, two major anti-cancer cytokines [28, 29]. After the K3-SPG administration, IL-12p40 and IFN-β were detected in the tumor microenvironment by immunohistochemical staining (Suppl Figures 5, 6). In order to clarify the role of IL-12 and type-I IFNs in the induction of tumor cell death and subsequent anti-tumor immune responses, we examined tumor cell death induction in mice lacking both IL-12 p40 and type-I IFN receptor. Tumor cell death characterized by CD45 negative dead tumor cells in the spleen after intravenous K3-SPG treatment were significantly reduced in *Il12p40* and *Ifnar2*-doubly deficient mice (Figure 5A, 5B). These results suggested that K3-SPG monotherapy induced both IL-12 and type-I IFN, and that these cytokines are required for the induction of immunogenic tumor cell death. Next, we examined the roles of IL-12 and type-I IFN *in vivo* in anti-tumor effect of K3-SPG. *Il12p40* and *Ifnar2*-deficient mice were inoculated with EG7 and i.v. administrated with PBS or K3-SPG at day 7, 9, 11. The anti-tumor effect of K3-SPG was partially reduced in mice lacking *Ifnar2*, but not in mice lacking *Il12p40* (Figure 5C, 5D). By sharp contrast, *Il12p40-* and *Ifnar2*-doubly deficient mice failed to display anti-tumor effects of K3-SPG (Figure 5E). These data demonstrated that intravenous injection of K3-SPG induces production of both IL-12 and type-I IFN in the tumor microenvironment, which are required for immunogenic tumor cell death, and essential for K3-SPG-mediated tumor regression.

## DISCUSSION

Innate immune responses are important for cancer immunoediting, the process whereby immune cells protect against cancer formation [5, 30]. We show the possibility of a new cancer immunotherapy by a nanoparticulate CpG. Our results suggest that phagocytes in the tumor microenvironment such as macrophages and dendritic cells engulfed K3-SPG, produce anti-cancer cytokines such as IL-12 and type-I IFN, potentially contributing to such cancer immunoediting. It is also conceivable that these cytokines may activate antigen-specific anti-tumor immune responses, as IFN-β in the tumor microenvironment can attract intratumoral dendritic cells, and expand cytotoxic T-lymphocytes by increasing antigen cross-presentation within the tumor microenvironment [31, 32]. Consistently, our results indicated that tumor specific CD8^+^ T cells were localized in the tumor microenvironment, which induced by intravenous K3-SPG injection (Suppl Figure 7), and these cells were sufficient for tumor regression as anti-CD8- antibody as well as absence of *Batf3* genes lacking CD8^+^ DCs abrogates anti-tumor effects (Suppl Figures 8A, 8B).

Intravenous K3-SPG treatment may be an effective anti-cancer mono-therapeutic agent by targeting tumor microenvironment and by activating both innate and adaptive immune pathways, regardless of tumor origin, location as well as immune milieu of tumor microenvironment. Uniqueness of K3-SPG as a mono-immunotherapeutic agent may be two fold, as the tumor antigen-specific CD8^+^ T cell-responses were induced and probably broaden by immunogenic tumor cell death with immune activation. While there are a variety of mechanisms involved in how tumor cells evades anti-tumor immunity as a barrier to cancer immunotherapy [5, 28, 33-36], K3-SPG is able to overcome one of these, at least in part by K3-SPG-induced immunogenic tumor cell death, potentially increasing neo- and/or subdominant tumor antigen-specific CD8^+^ T cells responses. Immunogenic tumor cell death induced by K3-SPG might be caused by activated phagocytes (macrophages and DCs) in the tumor microenvironment through IL-12 and type-1 IFN. In fact, K3-SPG anti-tumor effect was dependent on tumor resident phagocytes (Figure 3C). These results suggested that systemic administration of immunostimulatory agents such as TLR agonists that specifically target tumor microenvironment can overcome hurdles of current cancer vaccination with tumor antigen(s) as well as local immunotherapies. However, more detailed characterization of this critical population of tumor resident phagocytes in terms of their phenotypes and functions are required in the future study. In addition, although CD8^+^ DCs is essential to induce CD8^+^ T cells responses with our K3-SPG therapy, it needs future studies to investigate whether the tumor phagocytes with K3-SPG directly present tumor antigen to the CD8^+^ T cells.

Recently, immune checkpoint blockade antibody therapy is a big topic in cancer immunotherapy [1, 2]. Current topic is to find out the counter part of the immune checkpoint inhibitor, which synergize to build a more effective anti-tumor immunity. In that sense, K3-SPG is a promising cancer immunomodulator in combination with the immune checkpoint inhibitor, because K3-SPG activates both innate and adaptive immunity.

## MATERIALS AND METHODS

### Animals and reagents

Six-week-old C57BL/6J mice were purchased from CLEA Japan, Inc. *Il12 p40-* and *Batf3*- deficient mice were purchased from Jackson Laboratory. *Ifnar2-*deficient mice were previously described [37]. GFP transgenic mice (C57BL/6-Tg (CAG-EGFP) C14-Y01-FM131Osb) were obtained from Dr. M. Okabe (Osaka University). IFN-β-GFP KI mice were obtained from Dr. Y. Kumagai and Dr. S. Akira (Osaka University). All animal experiments were conducted in accordance with the institutional guidelines of the National Institutes of Biomedical Innovation, Health and Nutrition. The CpG oligodeoxynucleotides (ODNs) and the preparation and characterization of K3-SPG were previously described [10]. Ovalbumin (OVA) was purchased from Seikagaku Kogyo, Japan.

### Cell lines

EL4 and OVA-expressing EL4 (EG7) cells are thymoma cell lines from C57BL/6J mice, which were purchased from ATCC. B16 (melanoma) was purchased from the Japanese Collection of Research Bioresources. B16F10 (melanoma) was purchased from the RIKEN Cell Bank, and MC38 (colon cancer) was kindly provided by Dr. F. James Primus. Pan02 (pancreatic cancer) was purchased from the Jackson Laboratory. EG7, MC38, and Pan02 were cultured in complete RPMI (RPMI 1640 supplemented with 10% (v/v) fetal bovine serum (FBS), penicillin, and streptomycin). B16 and B16F10 were cultured in complete DMEM (DMEM supplemented with 10% (v/v) FBS, penicillin, and streptomycin).

### Tumor experiments and methods of therapy

EG7, B16, B16F10, and MC38 cells (100 μl of 5×10^6^ cells/mL in 10% Matrigel/PBS) were inoculated subcutaneously (s.c.) into the right flank of mice. The tumor size was measured by length (L), width (W), and height (H), and tumor volume (V) was calculated as V = L×W×H. Intratumoral injection (i.t.) was the direct injection into the tumor site. Intradermal injection (i.d.) was performed at the base of the tail on the same side as the tumor inoculation. CpG therapy was initiated when the tumor volume was 100 mm^3^ (day 7 after inoculation for EG7 and B16F10; day 10 after inoculation for B16; and day 14 after inoculation for MC38). The tumor-bearing mice were treated with K3 (30 μg) or K3-SPG (10 μg) 3 times every other day.

### Peritoneal dissemination model of pancreatic cancer by Pan02 cell line

To establish a peritoneal cancer dissemination model, pancreatic cancer cell line Pan02 (1×10^6^, 100 μl of 1×10^7^ cells/mL in PBS) was injected intra-peritoneally. CpG therapy was started at day 11 after inoculation, and all tumor nodules were resected from the peritoneum of mice at day 21, and then measured tumor's weight. The dosage of CpG therapy is described above.

### *In vivo* imaging experiments

To evaluate the localization of K3 and K3-SPG, C57BL/6 mice were inoculated with EG7 s.c. into the right flank at day 0 and were administered PBS, Alexa647-K3 (30 μg), or Alexa647-K3-SPG (10 μg) i.v., at day 12. One hour after administration, mice were analyzed by IVIS^Ò^ Lumina Imaging System and analysis software (Ver.2.6, Xenogen), in which images measured in relative fluorescence were converted into physical units of surface radiance (photons/sec/cm^2^/sr).

### Immunohistochemistry

C57BL/6J mice bearing EG7 in the right flank were administered with Alexa647-K3 (30 μg), Alexa647-K3-SPG (10 μg), or dextran-PE (20 μg) i.v. in the tail vein. Tumors were collected at 1 hour after injection, and frozen sections were fixed in 4% (w/v) paraformaldehyde for 10 min, and stained with anti-CD3e antibodies (BD Biosciences Pharmingen), and stained by anti-F4/80 (Biolegend), CD11b (Biolegend), anti-IL12-p40 (Biolegend), Streptavidin-PE (Biolegend), or anti-CD8β (Biolegend) antibodies, together with Hoechst 33258 (Life technologies). Cells were imaged using the Olympus IX81 system. Imaging data were analyzed by MetaMorph.

### *In vivo* depletion of phagocytes

To deplete phagocytes (dendritic cells and macrophages), C57BL/6J mice were injected i.v. with 200 μl of clodronate liposomes or control liposomes (100 nm) (Katayama Kagaku) at day 5 after inoculation with EG7. To deplete CD8 T cells, 200 μg of anti-CD8α antibody (purified from 53-6.7 hybridoma) was injected i.v. into the tail vein at day 6 and 13 after EG7 inoculation.

### Flow cytometric analysis

Splenocytes were collected at day 14 from EG7 bearing C57BL/6 mice or *Il12p40-Ifnar2* doubly deficient mice treated with PBS or K3-SPG i.v. at days 7, 9, and 11 or from naïve mice. After preparation of splenocytes, red blood cells were lysed with ACK lysis buffer and the cells were maintained in complete RPMI. Splenocytes were stained with H-2K^b^ OVA tetramer (MBL), anti-CD8α (KT15), -TCRβ (H57-597), -CD62L (MEL-14), and -CD44 (IM7) antibodies, and 7-amino-actino-mysin D (7AAD). OVA tetramer^+^ CD44^+^ CD8α^+^ TCRβ^+^ cell numbers were determined by flow cytometry.

Splenocytes were collected at day 12 from EG7 bearing C57BL/6 mice or *Il12p40-Ifnar2* double deficient mice treated with PBS or K3-SPG i.v. or with K3-SPG i.d. or with K3 i.t. at days 7, 9, and 11 or from naïve mice. After preparation of splenocytes, red blood cells were lysed with ACK lysis buffer and cells were maintained in complete RPMI. Splenocytes were stained with anti-CD45 antibody (BioLegend), and CD45 negative cell numbers were determined by flow cytometry. In addition, the populations of apoptotic, necrotic, and live CD45 negative cells were stained using propidium iodide (Life Technologies) and Hoechst 33342 (Life Technologies), and then analyzed by flow cytometry. CD45 negative cells were sorted from tumor bearing C57BL/6 mice treated with K3-SPG by INFLUX (BD Bioscience).

### Vaccination model

C57BL/6 mice were i.v. administered with 5×10^5^ CD45 negative cells at day −7. Seven days after immunization, mice were inoculated s.c. with 5×10^5^ EG7 cells.

## SUPPLEMENTARY MATERIALS FIGURES


