# Supplementary material for: Circulating nano-particulate TLR9 agonist scouts out tumor microenvironment to release immunogenic dead tumor cells
Source: Oncotarget. 2016 Jul 1;7(31):48860–9. doi: 10.18632/oncotarget.10379 (PMC5226476; doi:10.18632/oncotarget.10379)
Supplement: Supplementary file 1 [file oncotarget-07-48860-s001.pdf]

Circulating nano-particulate TLR9 agonist scouts out tumor microenvironment to release immunogenic dead tumor cells

Supplementary Material

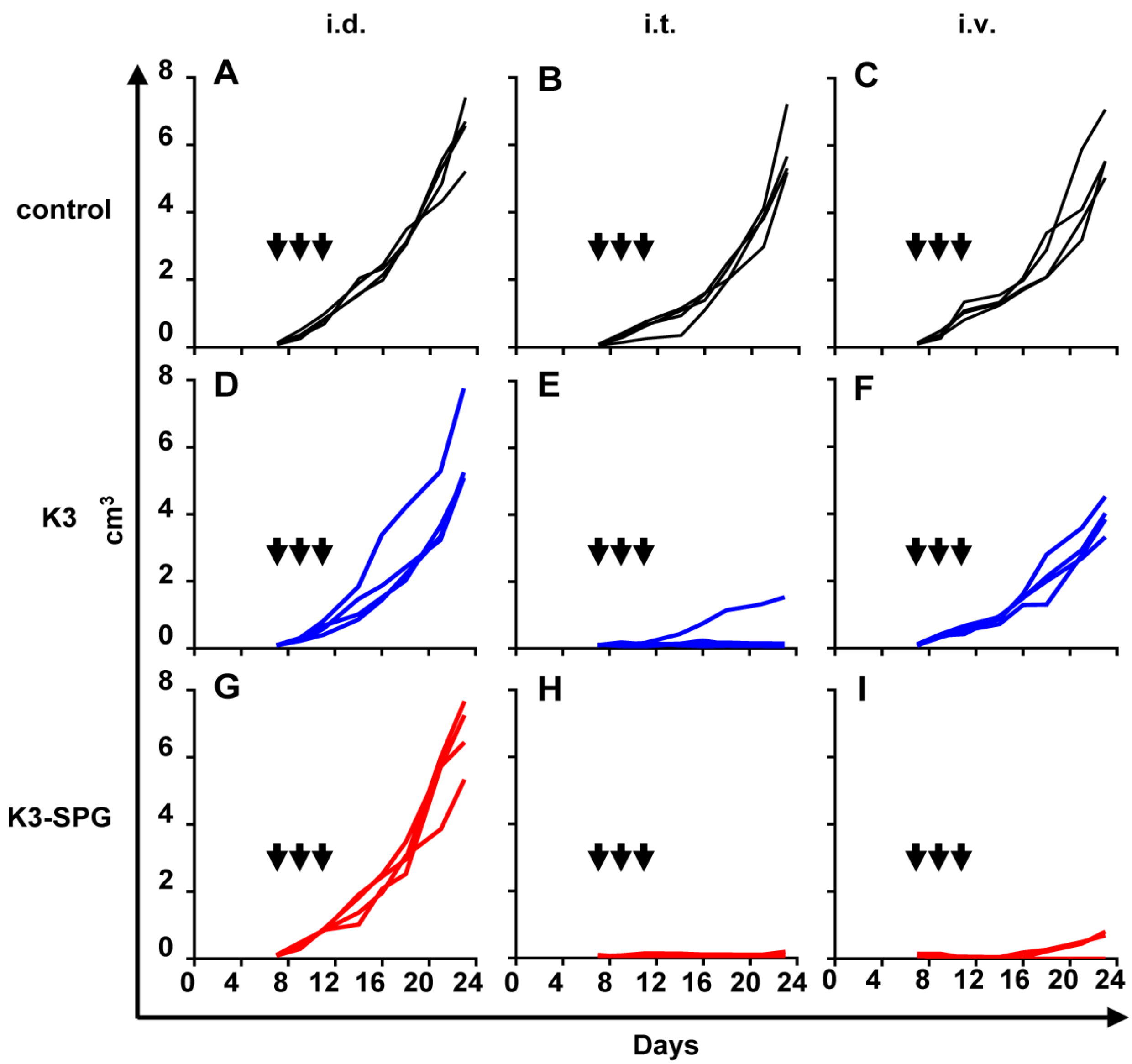

Supplementary Figure 1. K3-SPG i.v. without antigen can be applied for EG7 tumor model

C57BL/6 mice were inoculated s.c. with EG7 cells at day 0 and were administrated intradermally (i.d.) (peritumoral area) (**A**, **D**, **G**), intratumorally (i.t.) (**B**, **E**, **H**), or intravenously (i.v.) (**C**, **F**, **I**) with PBS (**A**-**C**), K3 (30 μg) (**D**-**F**), or K3-SPG (10 μg) (**G**-**I**) at day 7, 9, and 11. The size of tumor were was measured for 23 days (n=4). Each curve represents individual mouse. Arrows indicate timing of therapy

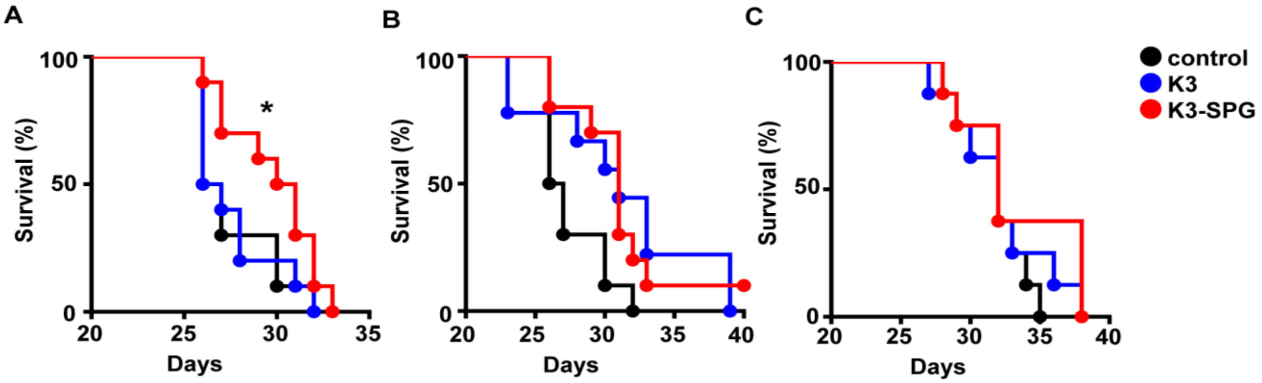

Supplementary Figure 2. The dose escalation test of systemic administration of K3 and K3-SPG without tumor antigen in pancreatic cancer peritoneum dissemination model C57BL/6 mice were i.p. injected with Pan02 at day 0 and were treated i.v. with PBS, K3 or K3-SPG three times. Survival rate (%) was representative (n=10). *$p < 0.05$ (Gehan-Breslow-Wilcoxon test). (**A**) K3 (10 μg) K3-SPG (10 μg) (**B**, **C**) K3 (50 μg) K3-SPG (50 μg)

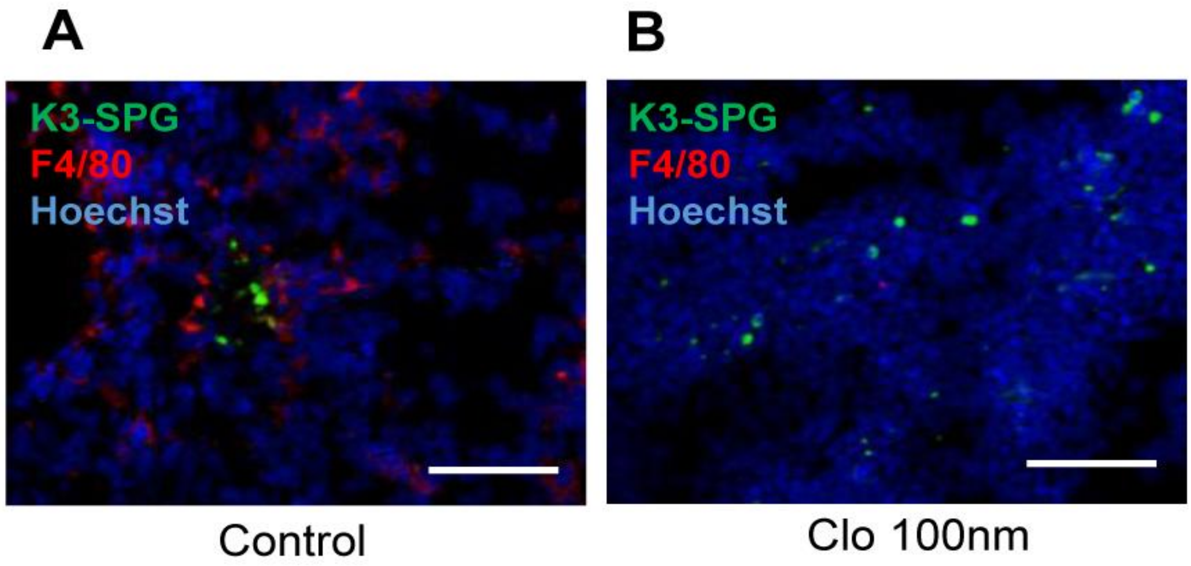

Supplementary Figure 3. F4/80 positive cells in the tumor were depleted by clodronate liposome

C57BL/6 mice were inoculated with EG7 at day 0 and administrated i.v. with liposome (**A**) or liposome (**B**) day 5, and treated i.v. with Alexa 647-K3-SPG at day 7. One hour after treatment, the frozen section of tumor was stained by anti-F4/80 antibody (red) and Hoechst33258 (blue) and then analyzed by fluorescent microscopy. (scale bars, 100μm)

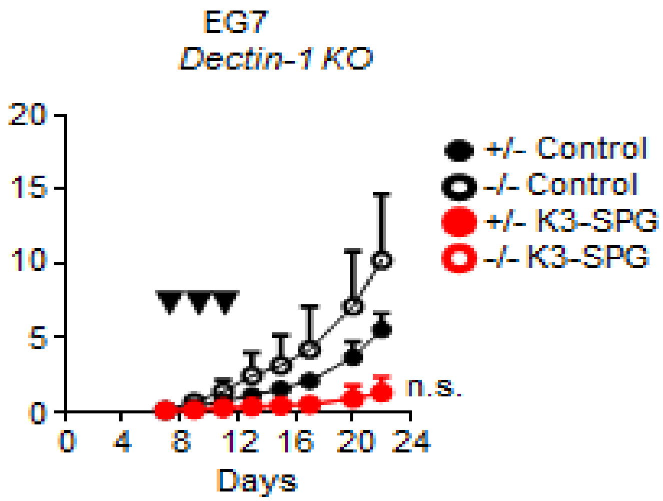

Supplementary Figure 4. Tumor regression effect of K3-SPG is not dependent on *Dectin-1* signal.

Dectin-1 KO and hetero mice were inoculated with EG7 at day0 and were treated with intravenous injection with K3-SPG at day7, 9, and11. n=5 (*Dectin-1*). Tumor growth is shown as mean + SEM. n.s.; not significant. $p < 0.05$ (t-test).

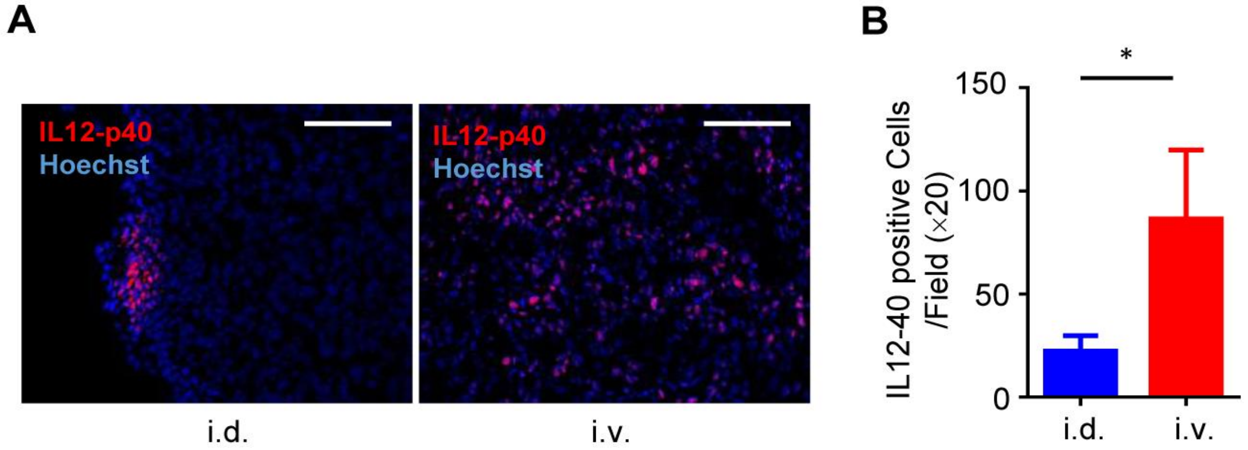

Supplementary Figure 5. IL12-p40 was detected in tumor-microenvironment

(**A**) C57BL/6 mice were inoculated with MC38 at day 0 and were treated i.d. or i.v. with K3-SPG at day 14, 16, and 18. Twelve days after inoculation, tumor was collected and the frozen section was stained by anti-IL12-p40 antibody (red)

and Hoechst 33258 (blue) and then analyzed by fluorescent microscopy. (scale bars, 100μm) (**B**) IL12-p40 positive cells were counted (10 fields each from 3 tumors). Error bars represent mean + SD. *$p < 0.05$ (*t*-test).

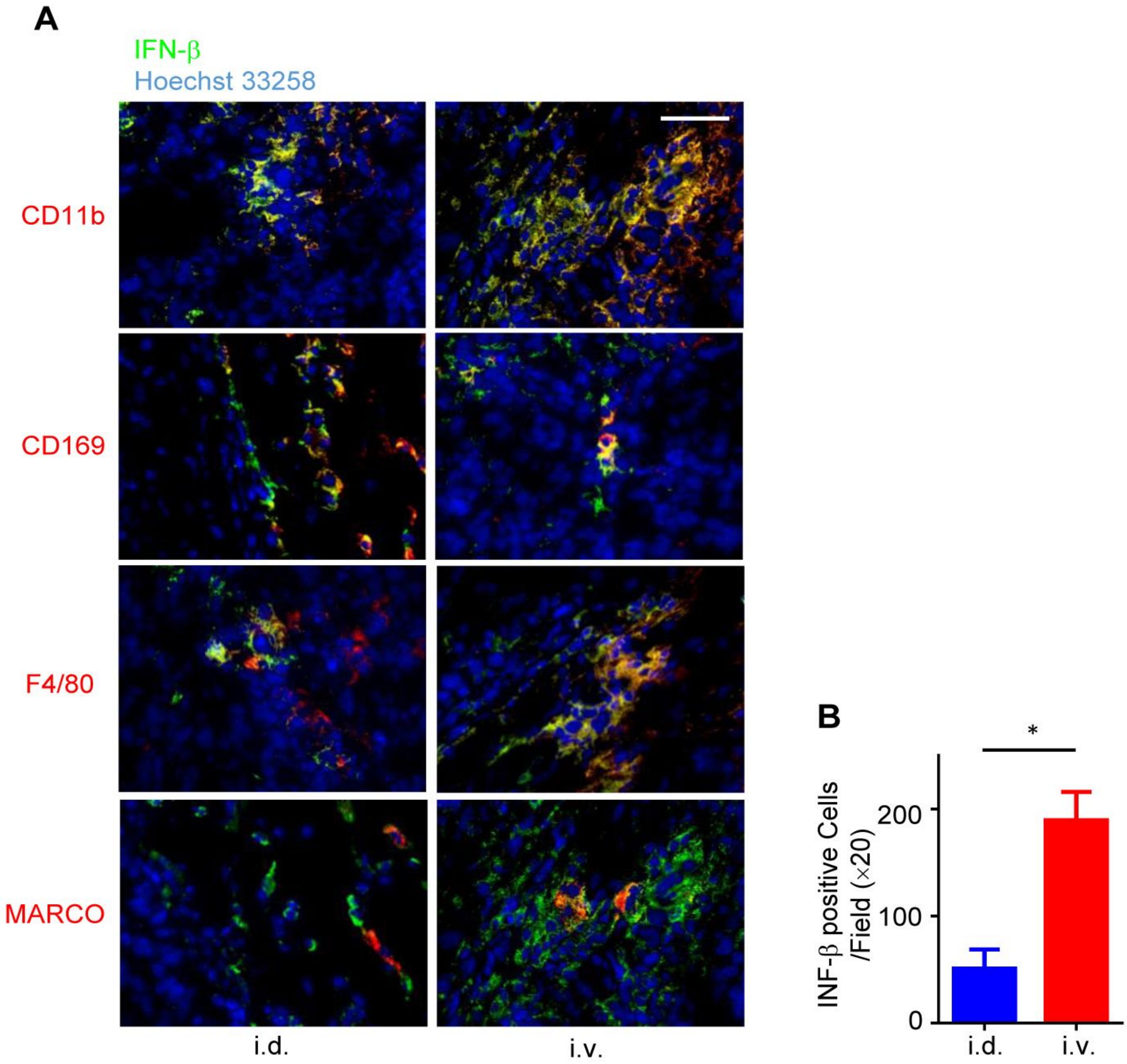

Supplementary Figure 6. IFN-β was detected in tumor-microenvironment

(**A**) *IFN-β* GFP mice were inoculated with MC38 at day 0 and were treated i.d. or i.v. with K3-SPG at day 14, 16, and 18. Twelve days after inoculation, tumor was collected and the frozen section was stained by anti-CD11b, CD169, F4/80, MARCO antibody (red) and Hoechst 33258 (blue) and then analyzed by fluorescent microscopy. (scale bars, 100μm) (**B**) *IFN-β* positive cells were counted (10 fields each from 3 tumors). Error bars represent mean + SD. *$p < 0.05$ (*t*-test).

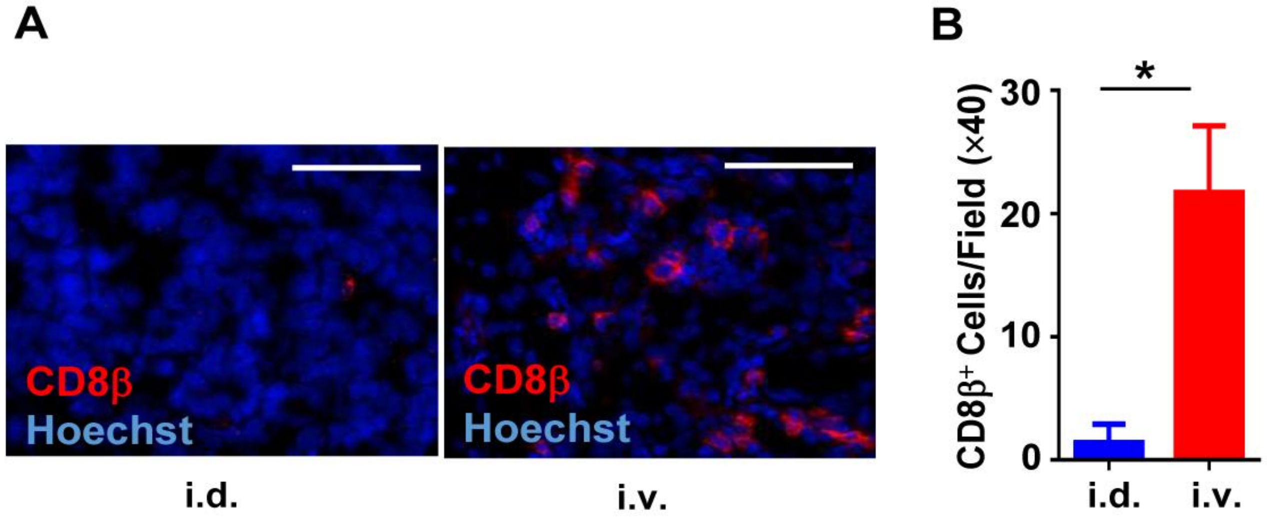

Supplementary Figure 7. CD8+ T-cells was detected in tumor microenvironment

(**A**)C57BL/6 mice were inoculated with EG7 s.c. at right flunk at day 0 and were treated with i.d. or i.v. with K3-SPG at day 7, 9, and 11. (**B**) At twelve days after inoculation, tumor was collected and the frozen section was stained by anti-CD8β antibody (red) and Hoechst 33258 (blue) and then analyzed by fluorescent microscopy. (scale bars, 100μm) CD8β positive cells were counted (10 fields each from 3 tumors). Error bars represent mean + SD. *p < 0.05 (*t*-test).

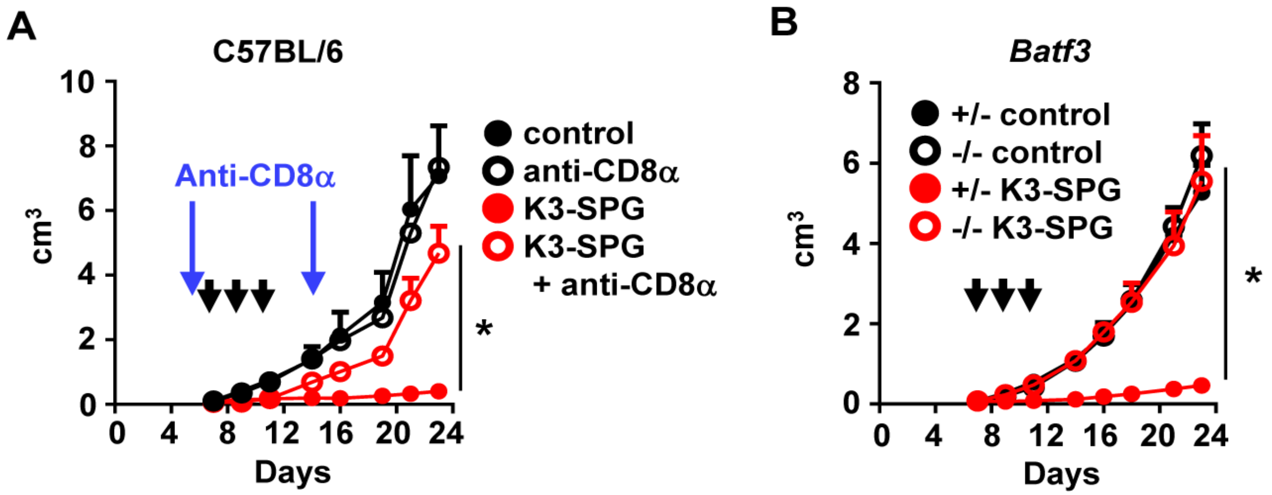

Supplementary Figure 8. K3-SPG-induced tumor regression requires immune responses

(**A**) C57BL/6 mice administered CD8α-depleting antibody (200 μg/mouse) or not at day 6 and 13, and were inoculated with EG7 s.c. at right flunk at day 0 and were treated with i.v. with K3-SPG at day 7, 9, and 11 (black arrow). (**B**) *Batf3* hetero and deficient mice were inoculated with EG7 s.c. at right flunk at day 0 and were treated with i.v. with K3-SPG at day 7, 9, and 11 (black arrow). Error bars represent mean + SEM (n=4). *$p < 0.05$ (t-test). Arrows indicate timing of therapy.
